# Posterior scleral deformations around optic disc are associated with visual field damage in open-angle glaucoma patients with myopia

**DOI:** 10.1371/journal.pone.0213714

**Published:** 2019-03-15

**Authors:** Eun Kyoung Kim, Hae-Young Lopilly Park, Chan Kee Park

**Affiliations:** 1 Department of Ophthalmology and Visual Science, College of Medicine,The Catholic University of Korea, Seoul, South Korea; 2 Seoul St. Mary’s Hospital, Seoul, South Korea; Federal University of São Paulo, BRAZIL

## Abstract

**Purpose:**

To identify important variables associated with visual field (VF) defects in open-angle glaucoma (OAG) with myopia.

**Materials and methods:**

A total of 105 OAG with myopia were enrolled in this cross-sectional study. The disc tilt ratio, disc torsion degree, disc-foveal angle, and area of peripapillary atrophy (PPA) were measured from red-free fundus photographs. Patients underwent Swept-source optical coherence tomography to measure peripapillary retinal nerve fiber layer (RNFL), subfoveal choroidal, and sufoveal scleral thicknesses. Functional evaluation was performed using 24–2 standard automated perimetry. For statistical analyses, logistic regression, artificial neural networks (ANN), and hierarchical cluster analysis were performed.

**Results:**

Logistic regression demonstrated peripapillary RNFL thickness as a significant variable for the presence of VF defects, otherwise ANN identified PPA area, peripapillary RNFL thickness, disc-foveal angle, and disc torsion degree as significant clinical variables in OAG with myopia. Two clusters were made after a hierarchical cluster analysis. Cluster 2 showed significantly worse VF damage than cluster 1 (MD = -5.20±5.25 dB for cluster 2 and -1.84±3.02 dB for cluster 1, P < .001). Cluster 2 had significantly greater disc tilt ratio, disc-foveal angle, and PPA area compared with cluster 1 (P < .001, 0.005, and < .001, respectively).

**Conclusions:**

Generally peripapillary RNFL thickness is considered as an important variable associated with visual field defects in glaucoma patients. ANN identified parameters associated with posterior scleral deformations around optic disc induced by myopic change including PPA area, disc torsion degree, and disc-foveal angle as significant clinical variables for visual field damage in OAG with myopia.

## Introduction

Myopia has been shown to be a risk factor for the development of glaucoma in meta-analyses of epidemiological surveys [1–3]. There have been numerous explanations for the possible relationship between myopic eyes and glaucoma, and the percentage of myopia, especially high myopia, is high in asian populations, it is important to better understand the relationships between myopia and glaucoma. However, glaucoma specialists have great challenges to evaluate glaucoma patients with myopia, since many of the optic discs of myopic patients are accompanied by posterior architecture deformation showing disc tilt, torsion, or peripapillary atrophy (PPA).

Another assault in myopic glaucoma patients is to interpret the discordance between optic disc change and visual field damage. In the clinic, we often face myopic glaucoma patients with visual field defects, even though they have small cup-disc ratio and retinal nerve fiber layer defect. To overcome this hurdle, we investigated the important clinical variables associated with visual field defects in open-angle glaucoma (OAG) patients with myopia. The visual field (VF), tested by standard automated perimetry, is a ubiquitous test of the visual function in glaucoma patients. Many researchers have tried to find significant ocular parameters associated with VF defects in glaucoma patients with myopia. Our group has reported that the direction of disc tilt and torsion could predict the location of glaucomatous VF defects in OAG eyes with myopia [4,5]. We also have published that posterior scleral thickness and PPA area are significantly related with glaucomatous VF defects in OAG eyes with myopia [6–8].

Advances in biostatistics and computing in the past several decades have led to creation of machine learning method of clinical prediction models. These include artificial neural networks and cluster analysis. These techniques complement classical or frequentist approaches, such as logistic regression analysis. Artificial neural networks mimic biological neural systems. In biological systems such as human brain and retina, incoming dendrites collect signals which are fed to the neuron. Artificial neural networks also take into account unobservable variables that the researcher is not aware of while designing the neural net [9–12].

In this regard, we designed this study to identify important clinical variables which have significant values to predict visual field damage in OAG patients with myopia using machine learning methods; artificial neural network and cluster analysis.

## Materials and methods

A total of 105 eyes of 105 OAG patients with myopia were enrolled in this cross-sectional study. We retrospectively reviewed the medical records of 105 patients by two glaucoma specialist (EKK, CKP) between December 2014 and August 2017 at the glaucoma clinic of Seoul St. Mary’s Hospital. The study was performed with the informed consent of the participants and followed all of the guidelines for investigation in human subjects required by the Institutional Review Board of Seoul St. Mary’s Hospital. All investigations were performed in accordance with the Declaration of Helsinki.

All open-angle glaucoma (OAG) patients underwent a comprehensive ophthalmologic examination, including measurement of best-corrected visual acuity, refraction assessment, slit-lamp biomicroscopy, Goldmann applanation tonometry, gonioscopic examinations, central corneal thickness via ultrasound pachymetry (Tomey, Nagoya, Japan), measurement of axial length using ocular biometry (IOL Master; Carl Zeiss Meditec, Dublin, California, USA), dilated stereoscopic examination of the optic nerve head, red-free RNFL photography (VX-10; Kowa Optimed, Tokyo, Japan), Swept-source OCT (DRI-OCT system; Topcon, Tokyo, Japan), and achromatic automated perimetry using the 24–2 Swedish Interactive Threshold Algorithm standard program (Humphrey Visual Field Analyzer; Carl Zeiss-Meditec, Inc., Dublin, CA, USA).

All patients were followed up every 1–3 months with color disc and fundus photography. VF and OCT examinations were performed at intervals of 6 months after the diagnosis of glaucoma.

Myopic patients with axial lengths > 24.0 mm in both eyes were evaluated in the present study. Among these myopic patients, glaucomatous VF defects corresponding to the Bjerrum area were eligible for the study. Eyes with VF defects were defined as having glaucomatous-appearing VF defects confirmed by at least 2 reliable VF examinations and the presence of a compatible glaucomatous optic disc that showed diffuse or localized rim thinning, a notch in the rim, a vertical cup-to-disc ratio > 0.7, or retinal nerve fiber layer (RNFL) defects.

Eyes had to meet the following additional inclusion criteria: best-corrected visual acuity ≥ 40/50, and presence of a normal anterior chamber and an open angle. Eyes with unreliable VFs (defined as false negative > 15%, false positive > 15%, fixation losses > 20%, and baseline MD less than −20 dB) were excluded. Patients with signs of pathologic myopia or myopic retinopathy (including posterior staphyloma, myopic choroidal neovascularization, lacquer crack, angioid streak) were excluded. Patients with intraocular or neurologic disease that could cause VF loss or with any retinal disease, such as diabetic macular edema, epiretinal membrane, or age-related macular degeneration were also excluded.

### Measurement of optic disc tilt ratio and torsion degree, disc-foveal angle, and PPA area

**Fig 1:**
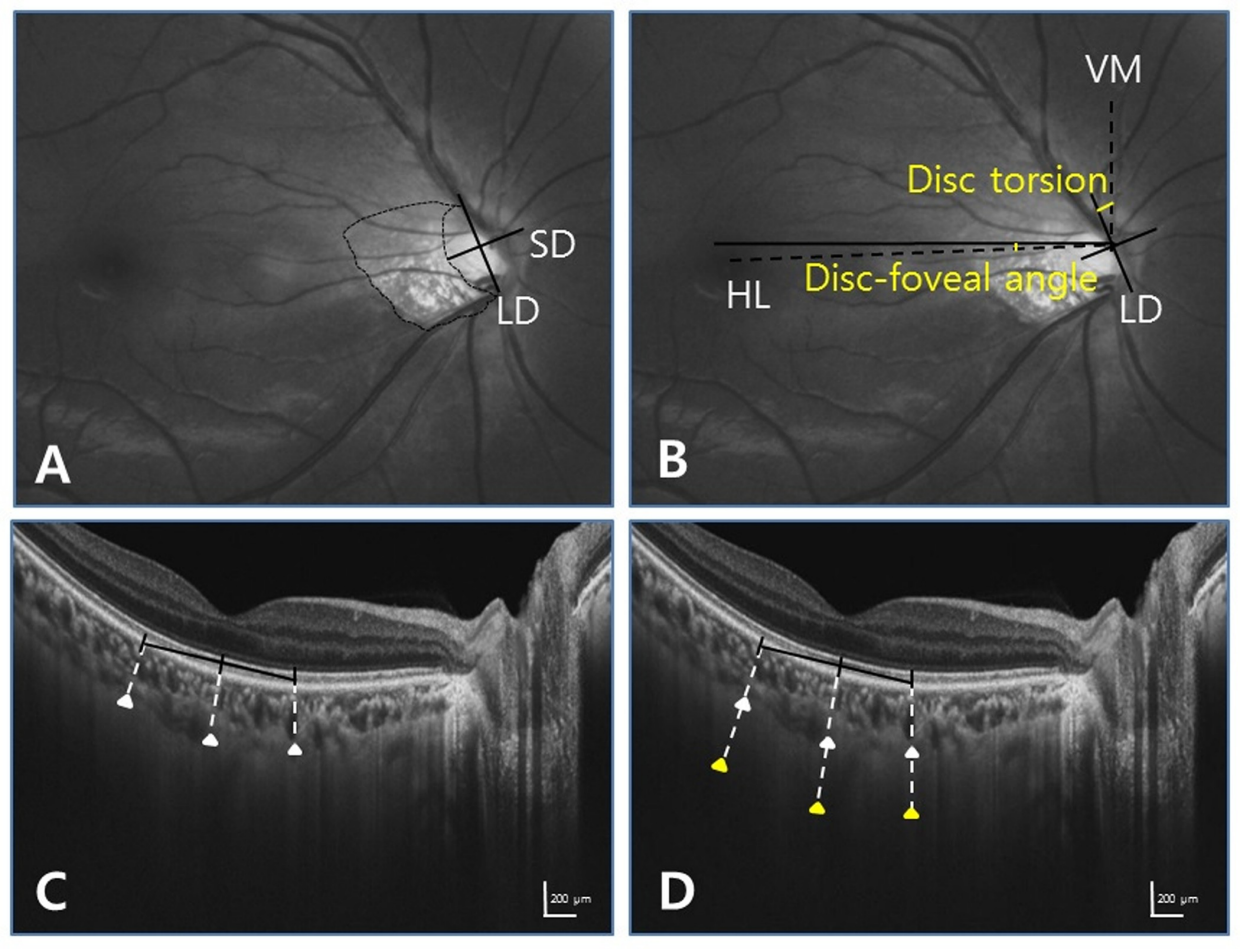
Identification of disc tilt ratio, torsion degree, disc-foveal angle, and peripapillary atrophy area by ImageJ software, and subfoveal choroidal and scleral thicknesses by the caliper function of the OCT software. Red-free fundus photographs (top), The horizontal cross-sectional images of the posterior pole obtained by Swept-source optical coherence tomography (bottom). (A) Tilt ratio was defined as the ratio between the longest diameter (LD) and shortest diameter (SD) of the optic disc. Peripapillary atrophy area was outlined manually, and the pixel area was calculated automatically using the software. (B) Torsion degree was measured the angle between the LD and vertical meridian (VM) which was considered a vertical line from a horizontal line (HL) connecting the fovea to the center of the optic disc. The disc–foveal angle was measured, defined as the angle between a horizontal line (HL) through the disc center and the line connecting the fovea and disc center. (C, D) Subfoveal choroidal and scleral thicknesses were measured at 3 locations: the subfoveal point and 500 mm temporally and nasally therefrom. Lamellated, high-reflective features are characteristic of the sclera. The full length of the sclera was visible with Swept-source OCT.

Digital retinal photographs centered on the optic disc and macular region were obtained using standardized settings. Optic disc tilt and torsion, disc-foveal angle, and PPA area were measured from red-free photographs by two observers (EKK, CKP) using the National Institutes of Health image analysis software (ImageJ 1.40; available from http://rsb.info.nih.gov/ij/index.html, National Institutes of Health, Bethesda, MD, USA) (Fig 1). Disc tilt was identified by the disc ovality ratio, defined as the ratio between the longest and shortest diameters of the optic disc. Disc torsion was identified and defined as the deviation of the long axis of the optic disc from the vertical meridian. The vertical meridian was considered a vertical line from a horizontal line connecting the fovea to the center of the optic disc. The angle between the vertical meridian and the longest diameter of the optic disc was considered the degree of torsion. A positive torsion value indicated infero-temporal torsion (which is counterclockwise torsion in the right eye format), and a negative value indicated supero-nasal torsion (which is clockwise torsion in the right eye format). The PPA area was plotted using a mouse-driven cursor to trace the disc and PPA margins directly onto the image. The pixel areas of the PPA were calculated using ImageJ software.

### Measurement of subfoveal choroidal and scleral thicknesses by Swept- source OCT

Choroidal thickness was defined as the vertical distance between the retinal pigment epithelium and the chorioscleral interface. Scleral thickness was defined as the distance between the chorioscleral interface and the outer scleral border. The outer surface of the sclera was carefully identified in OCT scans from retro-ocular structures. Scleral tissues were identified by their lamellar morphologic features and high reflectivity values. Using multi-averaged OCT images, we measured the choroidal and scleral thicknesses. Measurements were made using the caliper function of the OCT software by 2 masked observers (EKK, CPK). Choroidal and scleral thicknesses were measured at 3 points at the subfoveal region (Fig 1). The average of measurements at the subfoveal point and 500 mm temporally and nasally therefrom were calculated.

### Visual field measurements

All patients underwent VF testing using the Swedish Interactive Threshold Algorithm Standard (SAP) 24–2 strategy. A glaucomatous VF defect was defined as a cluster of 3 or more points with a probability <5% on the pattern deviation map, including at least 1 point with a probability of < 1%; or a result of outside normal limits in the glaucoma hemifield test; or a pattern standard deviation (PSD) with a probability of < 5%.

### Statistical analysis

Statistical analyses were performed using SPSS (Ver.23.0; SPSS, Chicago, IL, USA) and SAS software version 9.2 (SAS Institute, Cary, NC). Inter-observer reproducibility of the measurement of the center of optic disc and subfoveal scleral thickness was assessed, and the intraclass correlation coefficients (ICC) with 95% confidence intervals (CI) were calculated. In this study, at first, artificial neural networks (ANN) model was used. In the ANN modeling process, we randomly divided the data into two subsets: for constructing the models (training subset) and the remaining (nearly 40%) for testing the model (as the validation subset). After evaluating the model, we applied multiple layer perceptron (MLP) networks to determine important factors for glaucomatous visual field defects. In this context, we use independent variable importance analysis and by using normalized importance, the significant variables associated with visual field defects were determined. Secondly, logistic regression analysis was done to find clinical variables associated with visual field defects in OAG eyes with myopia. Lastly, a hierarchical cluster analysis was used to classify myopic glaucoma patients. Specifically, the agglomerative technique was performed to group OAG eyes with myopia into homogenous subgroups, which began with each eye being a cluster by itself and continued until similar clusters merged together. Ward’s minimum variance method was used as clustering criterion, and all variables were standardized before clustering. To determine k as the optimal number of clusters, 3 statistical values were examined: the biggest drop of semipartial R^2^ and pseudo t^2^ statistics at k clusters compared with k-1 clusters and maximum pseudo F statistic at k clusters. The student t test was used to compare continuous variables and the chi-square test to compare categorical variables between clusters. A value of P < .05 indicated statistical significance.

## Results

This study involved 105 OAG eyes with myopia. The demographics of subjects with open-angle glaucoma are summarized in Table 1. There were excellent agreement between two graders for measurements of variables. The intraclass correlation coefficients (ICCs) for the measurements of the center of optic disc and subfoveal scleral thickness were 0.961 (95% CI, 0.931–0.990) and 0.954 (95% CI, 0.934–0.986) respectively.

**Table 1:** Demographic and ocular characteristics of participants with glaucoma.

	Glaucoma
No. of subjects, eyes	105
Mean age, yrs	52.81±11.69
Gender, Male:Female	49:56
Intraocular pressure, mmHg	15.82±2.66
Central corneal thickness, μm	543.11±31.68
Axial length, mm	26.17±0.65
Visual acuity	0.96±0.10
Mean MD of 24–2 VF, dB	-3.90±4.66
Mean PSD of 24–2 VF, dB	5.82±4.88

Data are presented as the mean and standard deviation. MD = mean deviation; PSD = pattern standard deviation; VF = visual field.

### Logistic regression analysis

Variables for visual field defects in OAG with myopia included intraocular pressure, central corneal thickness, axial length, peripapillary RNFL thickness, disc tilt ratio, disc torsion degree, disc-foveal angle, PPA area, subfoveal choroidal thickness, and subfoveal scleral thickness. Dependent variable was the MD values of visual field exam. Peripapillary RNFL thickness was a significant clinical variable associated with visual field defects in OAG patients with myopia (P < .001) (Table 2).

**Table 2:** Logistic regression analysis demonstrates significant variables for visual field defects in open-angle glaucoma patients with myopia.

Variables	β	SE	t-statistics	*P* value
Intraocular pressure, mmHg	.085	.1050	.811	.419
Central corneal thickness, μm	.014	.009	1.480	.142
Axial length, mm	.337	.451	.747	.457
Peripapillary RNFL thickness, μm	.108	.022	4.87	.000
Disc tilt ratio	.995	2.145	.464	.644
Disc torsion degree	-.001	.015	-.053	.958
Disc foveal-angle, degree	.138	.084	1.634	.106
PPA area, pixel	.717	.723	.992	.324
Subfoveal choroidal thickness, μm	-.002	.005	-.418	.677
Subfoveal scleral thickness, μm	.000	.005	.015	.988

RNFL = retinal nerve fiber layer; PPA = peripapillary atrophy; β = estimated regression coefficient, SE = standard error.

### Artificial neural networks

**Fig 2:**
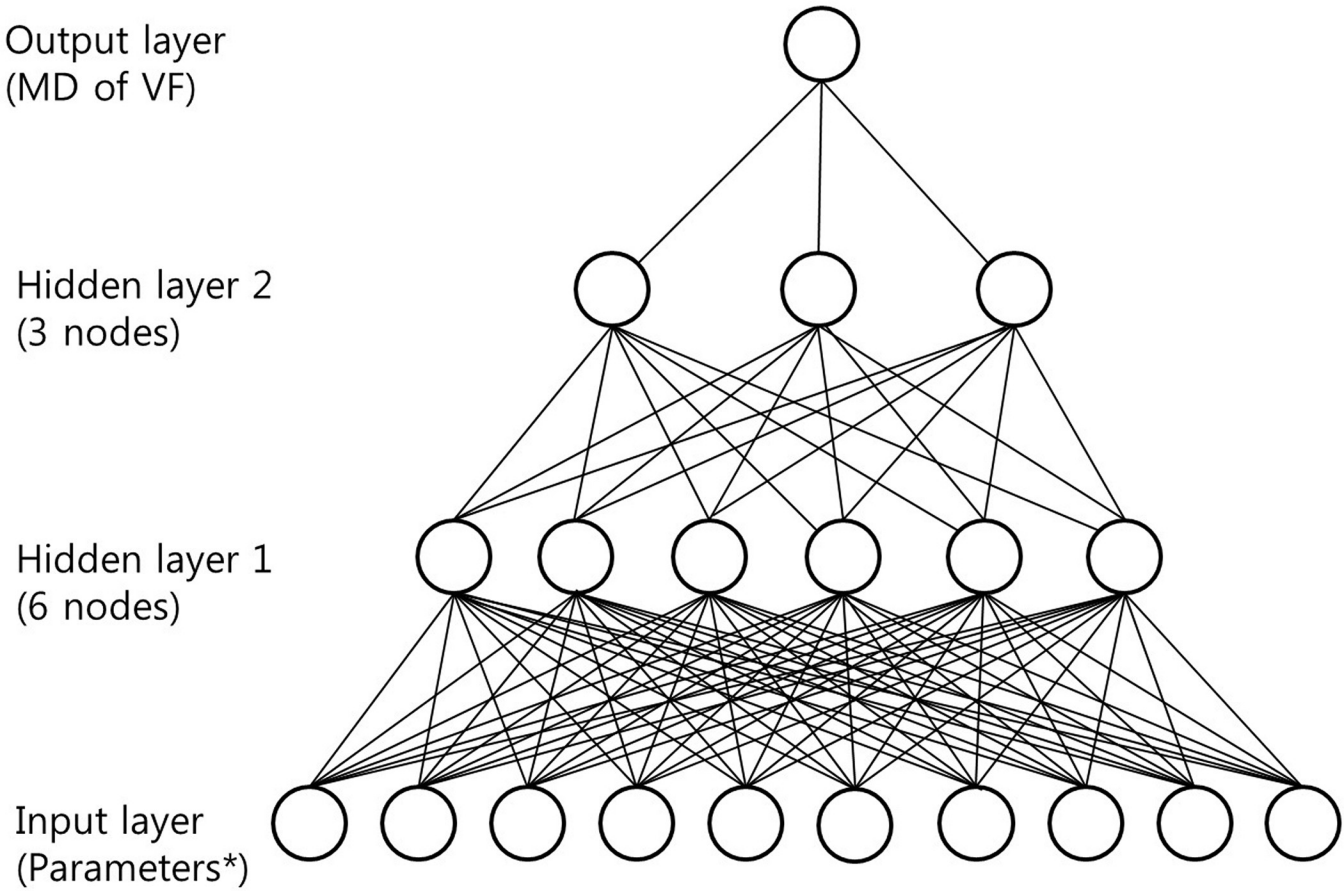
Diagram showing the structure of artificial neural networks used in the current study. In the multilayer perceptron, there were 10 nodes in the first input layer, 6 nodes in the second layer, and 3 nodes in the third layer. Each of the 4 layers (input, two hidden, and output layers) were connected through the stacked denoising autoencoder. Artificial neural network output diagram with insets for each layer. Output figure generated by IBM SPSS vesion.23.0 (Chicago, IL, USA). *parameters include 10 variables.

Artificial neural networks (ANN), created using IBM SPSS, for visual field defects in OAG patients with myopia using the database revealed the following features (Fig 2):

Model type: Multilayer Perceptron (MLP)
Number of layers: 4
Layer 1: 10 input variables
Layer 2: hidden layer, 6 hidden or latent variables
Layer 3: hidden layer, 3 hidden or latent variables
Layer 4: 1 output nodes
Training: 60% of sample size
Testing: 40% of sample size
Training algorithm: gradient descent

**Fig 3:**
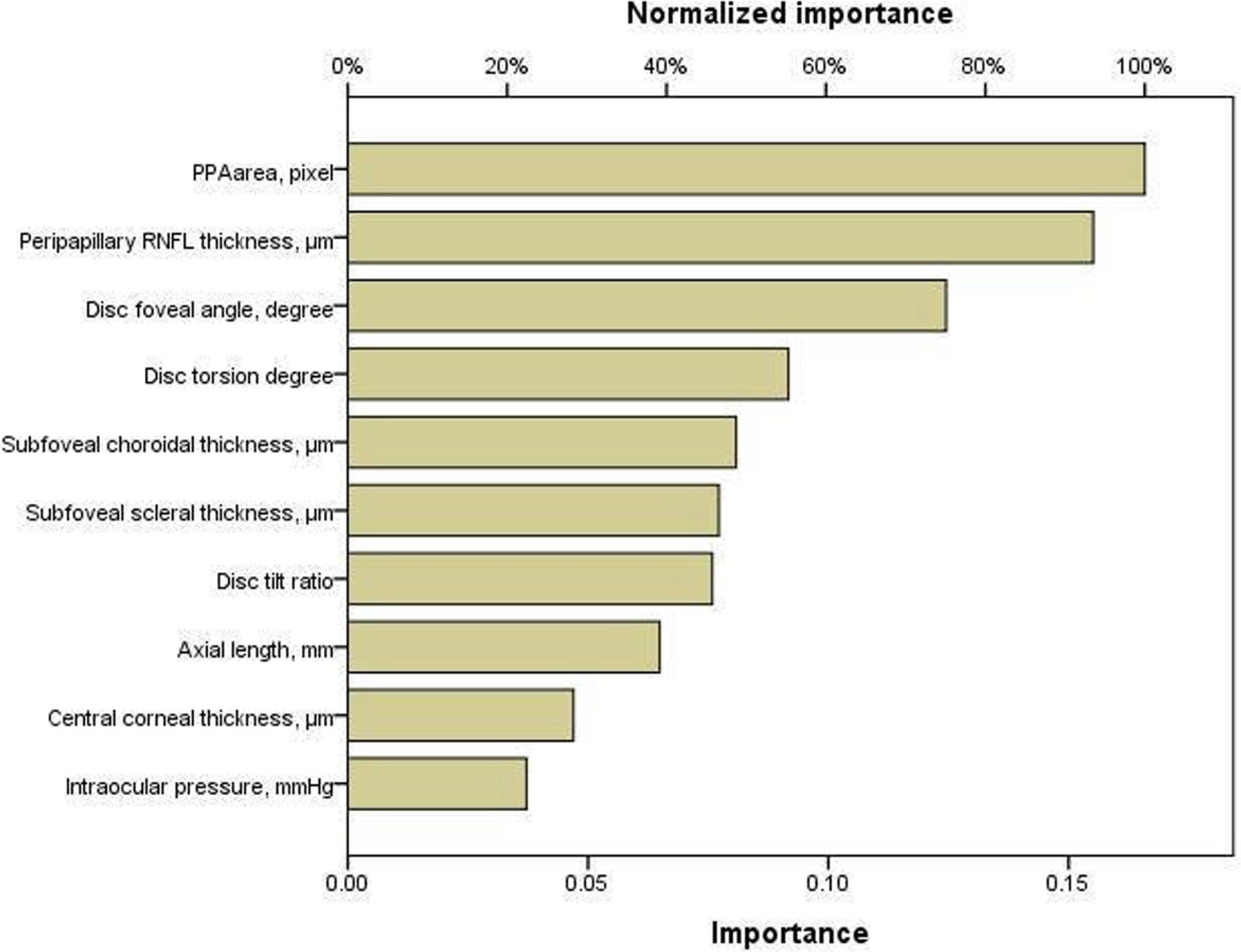
Results of artificial neural networks reveal importance and normalized importance values of ten variables for visual field defects in open-angle glaucoma patients with myopia. Output figure generated by IBM SPSS vesion.23.0 (Chicago, IL, USA). RNFL = retinal nerve fiber layer; PPA = peripapillary atrophy.

**Fig 4:**
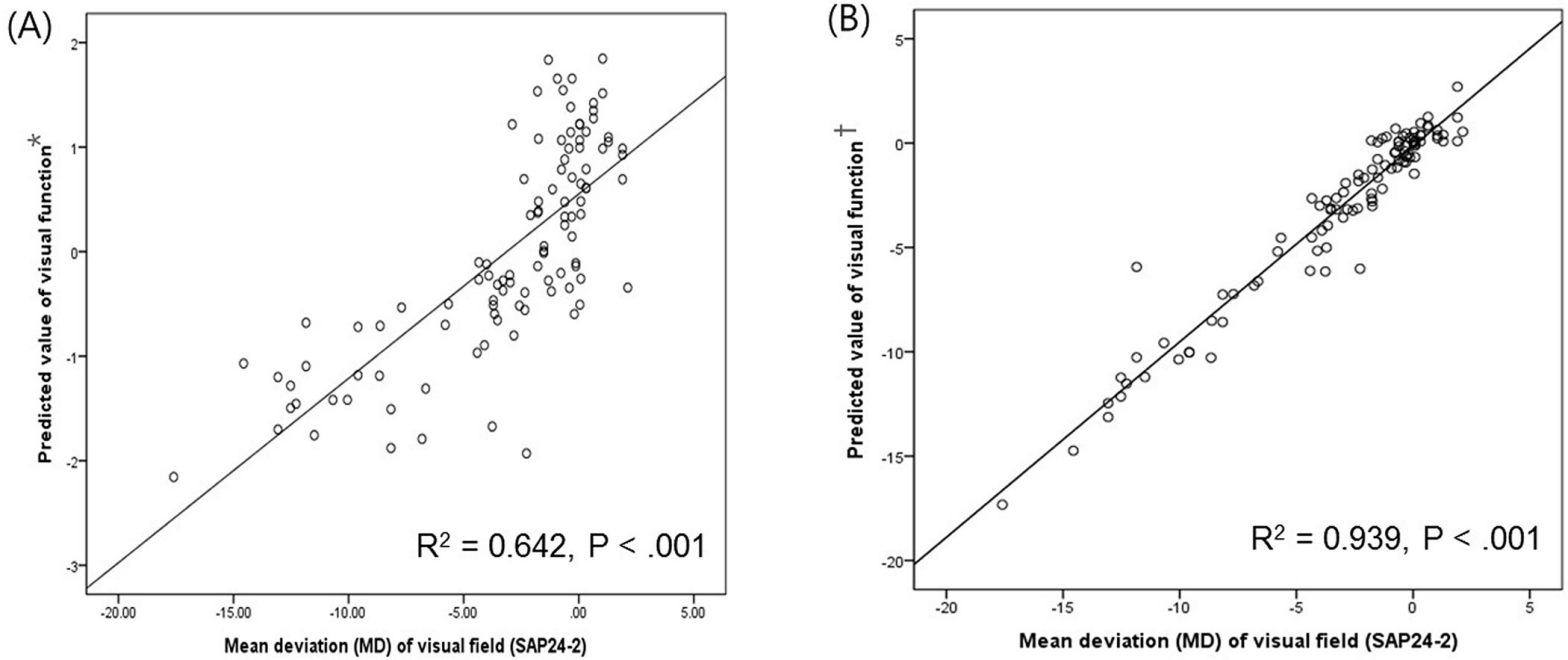
Scatterplots illustrating the linear correlation between standard automated perimetry (SAP) mean deviation (MD) value (dB) and predicted value of visual function. (A) Correlation between MD value of SAP and the predicted value of visual function using peripapillary RNFL thickness (R^2^ = 0.642, P < .001). (B) Correlation between MD value of SAP and the predicted value of visual function using 4 variables including PPA area, peripapillary RNFL thickness, disc-foveal angel, and disc torsion degree(R^2^ = 0.939, P < .001). *Predicted value of visual function was calculated using peripapillary RNFL thickness. ^†^Predicted value of visual function was calculated using PPA area, peripapillary RNFL thickness, disc-foveal angel, and disc torsion degree.

Variables, in order of magnitude of normalized importance in Table 3, include intraocular pressure, central corneal thickness, axial length, peripapillary RNFL thickness, disc tilt ratio, disc torsion degree, disc-foveal angle, PPA area, subfoveal choroidal thickness, and subfoveal scleral thickness. ANN identified PPA area, peripapillary RNFL thickness, disc-foveal angle, and disc torsion degree as significant clinical variables associated with VF defects in OAG eyes with myopia (Fig 3). Fig 4 shows the scatter plot of MD value of SAP and the predicted value of visual function. The predicted value of visual function using 4 parameters including PPA area, peripapillary RNFL thickness, disc foveal angel, and disc torsion showed greater R-squared value (R^2^ = 0.939, P < .001) compared with the predicted value of visual function using peripapillary RNFL thickness alone (R^2^ = 0.642, P < .001).

**Table 3:** Results of artificial neural networks reveal normalized importance values of variables for visual field defects in open-angle glaucoma patients with myopia.

Independent variable	Importance	Normalized importance
Intraocular pressure, mmHg	.037	22.4%
Central corneal thickness, μm	.047	28.3%
Axial length, mm	.065	39.1%
Peripapillary RNFL thickness, μm	.155	93.5%
Disc tilt ratio	.076	45.7%
Disc torsion degree	.092	55.3%
Disc-foveal angle, degree	.125	75.1%
PPA area, pixel	.166	100.0%
Subfoveal choroidal thickness, μm	.081	48.7%
Subfoveal scleral thickness, μm	.077	46.5%

RNFL = retinal nerve fiber layer; PPA = peripapillary atrophy.

### Hierarchical cluster analysis

**Fig 5:**
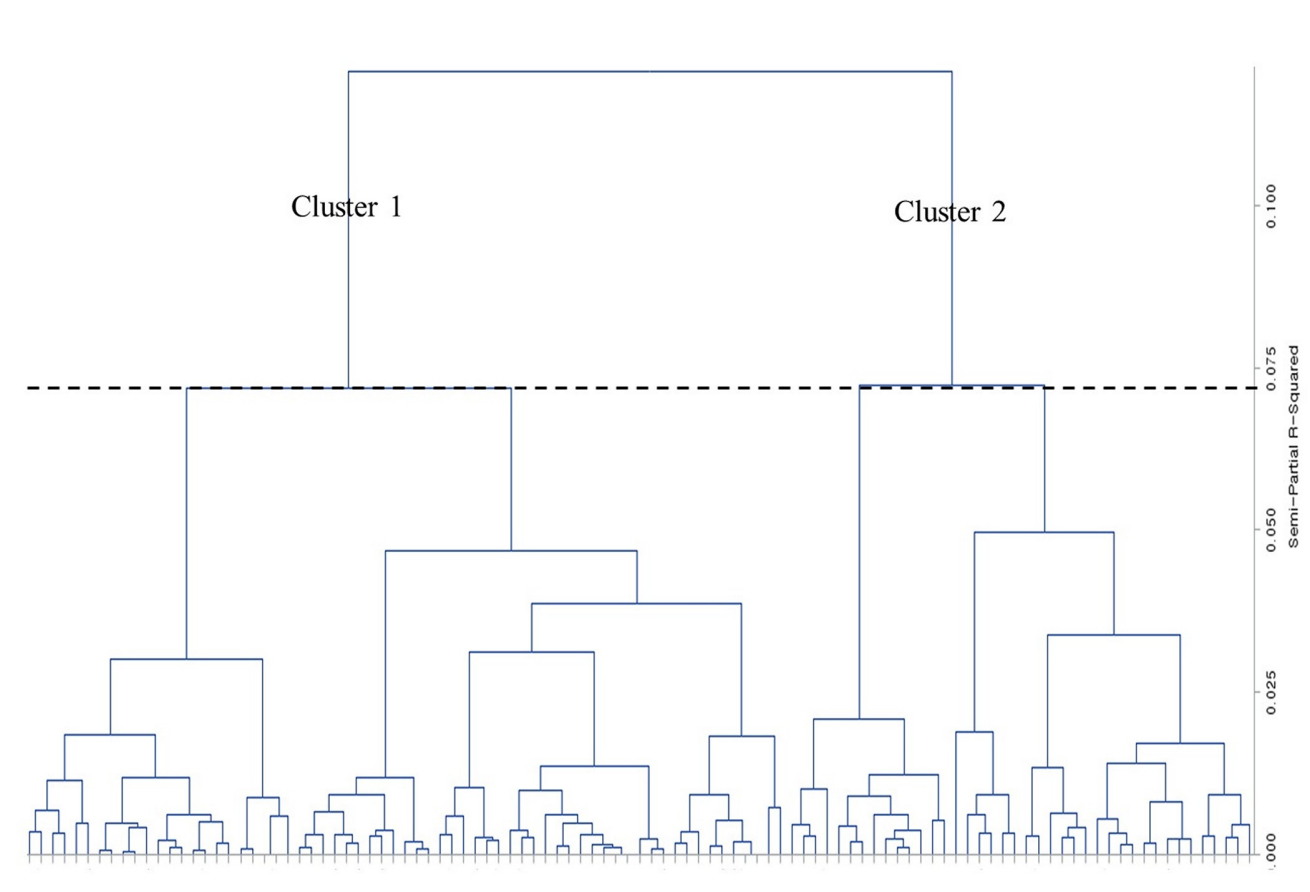
Dendrogram shows how clusters were made by the agglomerative technique, which began with each subject being a cluster by itself and merged together continuously based on similarity between clusters. The two-cluster solution in this analysis had a semipartial R^2^ value of 0.072, and two clusters seemed to best represent the data in this procedure. Output figure generated by Output figure generated by SAS software version 9.2 (SAS Institute, Cary, NC).

Using a hierarchical cluster analysis, all OAG patients with myopia were classified based on ten variables. To determine the number of clusters that best fit the data, we found the small semipartial R^2^ and pseudo t^2^ statistics followed by a much larger value. The two-cluster solution in this analysis had a semipartial R^2^ value of 0.072 and a pseudo t^2^ value of 8.7 followed by a value of .121 and 14.1 for a one-cluster solution. Thus, two clusters seemed to best represent the data in this procedure (Table 4 and Fig 5). Cluster 1 included 65 eyes and 40 eyes belonged to cluster 2. Comparisons of patient demographics and other variables between the two clusters are shown in Table 5. The eyes in cluster 2 showed significantly worse MD (-5.20±5.25 dB), greater disc tilt ratio and disc-foveal angle, and larger PPA area compared with cluster 1. However, there were no significant differences in age, sex, intraocular pressure, central corneal thickness, axial length, and either peripapillary RNFL thickness between two clusters.

**Table 4:** Statistical criteria to determine the optimal number of clusters.

No. Clusters	R^2^*	Semipartial R^2^^†^	Pseudo F Statistic^‡^	Pseudo t^2^ Statistic^§^
1	0	.121	0	14.1
2	.121	.072	14.1	8.7
3	.193	.072	12.2	10.8
4	.265	.050	12.1	6.6
5	.315	.047	11.5	7.8

*R^2^ measures the heterogeneity of the cluster solution formed at a given step. A large value signifies that clusters at a given step are different from each other.

^†^Semipartial R^2^ measures the loss of homogeneity due to merging of 2 clusters to form a new cluster at a given step. A small value suggests that the cluster solution obtained at a given step is formed by merging of 2 very homogenous clusters.

^‡^Pseudo F statistic describes the ratio of between cluster variance to within cluster variance. Peak in the pseudo F statistic is indicative of large cluster separation.

^§^Pseudo t^2^ statistic indicates the appropriate number of clusters. A potentially optimal number of clusters is indicated when a small pseudo t^2^ statistic is followed by a much larger value.

**Table 5:** Characteristics of the 2 clusters obtained by a hierarchical cluster analysis.

Variables	Cluster 1	Cluster 2	P value
No. of subjects, eyes	65	40	
Mean age, yrs	47.13±13.67	46.71±11.69	.866
Gender, male:female	29:24	27:25	.774^†^
Intraocular pressure, mmHg	14.15±3.12	15.02±2.78	.152
Central corneal thickness, μm	538.03±34.98	539.10±32.33	.144
Axial length, mm	26.02±0.68	26.41±0.70	.364
Peripapillary RNFL thickness, μm	76.25±12.44	84.97±19.91	.058
Disc tilt ratio	1.12±0.09	1.17±0.12	< .001
Disc torsion degree	17.41±15.11	21.29±19.65	.113
Disc foveal angle, degree	6.80±3.50	7.54±4.78	.005
PPA area, pixel	8192.06±5588.96	13813.77±7645.48	< .001
Subfoveal choroidal thickness, μm	227.81±71.73	226.69±39.12	.511
Subfoveal scleral thickness, μm	325.44±52.83	334.82±78.15	.996
MD of visual field, dB	-1.84±3.02	-5.20±5.25	< .001

RNFL = retinal nerve fiber layer; PPA = peripapillary atrophy; MD = mean deviation.

^†^ Comparison between the two groups by chi-square test.

## Discussion

Evaluating glaucoma in myopia poses great challenges for glaucoma specialists. The main aims of this study were identifying important clinical variables associated with visual field defects in OAG patients with myopia. In this study, artificial neural networks (ANN) strategy identified PPA area, peripapillary RNFL thickness, disc-foveal angle, and disc torsion degree as important clinical variables of visual field defects in OAG patients with myopia. Additionally we performed a hierarchical cluster analysis to subdivide OAG patients with myopia, and disc tilt ratio, disc-foveal angle, and PPA area were identified as significant variables to separate clusters. Collectively, our results suggest that variables of posterior scleral deformations around optic disc induced by myopic change are significantly associated with visual field damage in OAG patients with myopia

In ANN strategy, PPA area was the most important variables associated with visual field defects of myopic glaucoma patients in order of magnitude of normalized importance (Fig 3). Numerous studies reported that a larger PPA area was related to glaucomatous damage and progression of glaucoma [13–16]. PPA develops as the axial length elongates in early adulthood, and structural weakness resulting from myopic changes around the optic nerve head may contribute to glaucomatous damage. In addition to structural weakness, PPA causes blood flow insufficiency around optic nerve head possibly aggravating glaucomatous damage [17,18]. The most of recent studies dealing with the relationships between PPA and glaucoma have focused on β-zone area by the benefit of advances in imaging equipment and software [19,20]. The β-zone PPA is the PPA without the presence of retinal pigment epithelium and can be detected using optical coherence tomography. In our study, we did not include β-zone PPA in the analysis, however we found significant association between total PPA area which was easily measurable with fundus photography and glaucomatous VF defects in myopic glaucomatous eyes.

In this study, disc-foveal angle and disc torsion degree were significant clinical variables for the presence of VF defects in OAG with myopia by artificial neural networks. Structural and morphologic changes of the optic disc owing to axial elongation are thought to be important in the development and progression of glaucoma in myopic eyes. Disc tilt, disc torsion, and increased disc-foveal angle are typical features of myopic eyes and have been reported to be related to the location of glaucomatous damage in myopic eyes [21–24]. These myopic peripapillary scleral changes may result in the fragility of the supporting tissue in the lamina cribrosa and dynamic imbalances of the optic nerve head.

We also performed a hierarchical cluster analysis to classify myopic OAG patients according to the features of the optic disc and peripapillary sclera. A hierarchical cluster analysis is a useful statistical method to classify disorders by grouping subjects into homogenous subgroups based on similar characteristics. The eyes in cluster 2 showed significantly worse visual field defects, greater disc tilt ratio and disc-foveal angle, and larger PPA area compared with cluster 1 (Table 5). These results imply that eyes with posterior scleral deformations around optic disc induced by myopic change have worse visual field damage in glaucoma.

Some researchers suggests that myopic open-angle glaucoma, especially normal tension glaucoma, should be considered to be a combination of myopic optic neuropathy and glaucomatous optic neuropathy [25,26]. Myopia directly affects the lamina cribrosa (LC) by causing LC structural weakening [27]. Posterior scleral deformations such as progressive tilting of the optic disc and enlargement of PPA occurred during myopic shift might increase susceptibility for glaucomatous optic nerve fiber loss [28]. Furthermore, a sharp angle of scleral bending causes direct damage to the retinal nerve fibers, and leads to VF defects in myopic eyes [29]. Our group also have published several studies to support the idea that myopic optic neuropathy and glaucomatous optic neuropathy have a common denominator [4–8, 21, 22]. In this study, we could confirm the significant association between posterior scleral deformations and the presence of VF damage in myopic glaucoma patients.

There were some limitations of the present study. Patients were studied retrospectively and selection bias was not fully excluded. The cross-sectional design also had limitations, because we could not observe causal relationships nor definitively determine the order of results. Another limitation is that a greater number of subjects increase the accuracy of machine learning analysis. For further study, we plane to include more individuals for proper analysis.

In conclusion, an important contribution of this study was that for the first time we identified important clinical variables associated with visual field (VF) defects using machine learning methods in open-angle glaucoma (OAG) patients with myopia. We suggested that posterior scleral deformations around optic disc induced by myopic change might have noteworthy values for visual function prediction. Our study also revealed that myopic glaucomatous eyes had two distinct types characterized by features of posterior scleral deformations, and these myopic scleral changes significantly influenced the visual field damage.

## Supporting information

S1 Fig Images showing the measurement of subfoveal choroidal and scleral thickness in myopic glaucoma pateints by the caliper function of the OCT software. The average of measurements at the subfoveal point and 500 μm temporally and nasally therefrom were calculated.
(TIF)
