# Supplementary figures and images for: Posterior scleral deformations around optic disc are associated with visual field damage in open-angle glaucoma patients with myopia
Source: PLoS One. 2019 Mar 15;14(3):e0213714. doi: 10.1371/journal.pone.0213714 (PMC6420008; doi:10.1371/journal.pone.0213714)

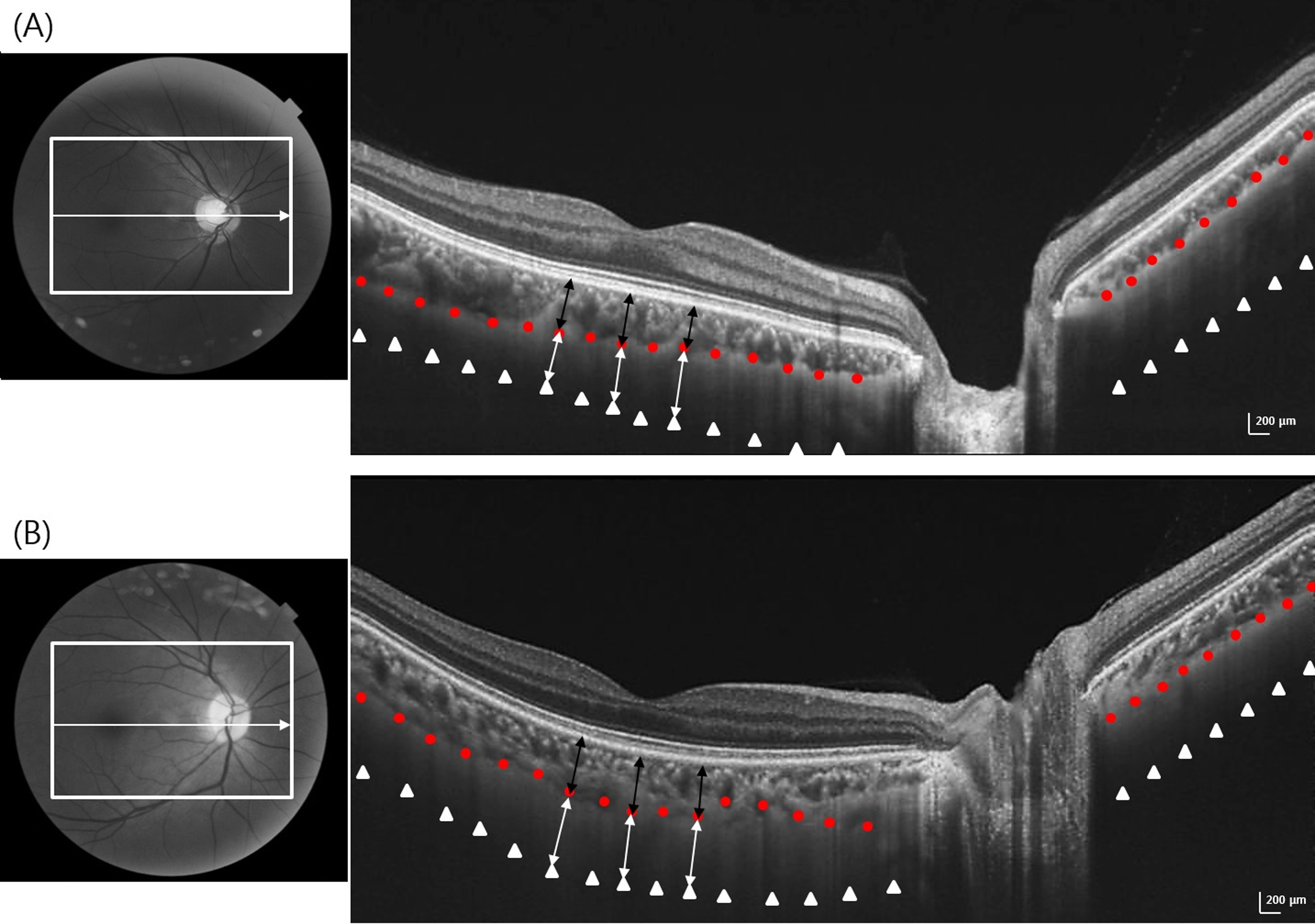

Supplement: S1 Fig — The average of measurements at the subfoveal point and 500 μm temporally and nasally therefrom were calculated. (TIF) [file pone.0213714.s001.tif]
